# Functional residual capacity tool: A practical method to assess lung volume changes during pulmonary complications in mechanically ventilated patients

**DOI:** 10.4103/0972-5229.74175

**Published:** 2010

**Authors:** S. Veena, Sudeep Palepu, G. S. Umamaheswara Rao, V. J. Ramesh

**Affiliations:** **From:** Department of Neuroanaesthesia, National Institute of Mental Health and Neurosciences, Bangalore, India

**Keywords:** Alveolar recruitment, functional residual capacity, mechanical ventilation, monitoring, positive end expiratory pressure, pulmonary complications

## Abstract

In this report, we describe a patient in whom we used a functional residual capacity (FRC) tool available on a critical care ventilator to identify the loss of lung volume associated with pulmonary complications and increase in FRC with the application of a recruitment maneuver. The case report underlines the utility of the FRC tool in rapid visualization of the lung volume changes and the effects of application of corrective strategies in patients receiving mechanical ventilation.

## Introduction

Loss of lung volume resulting in hypoxia is characteristic of acute lung injury (ALI).[1] A bedside tool for measuring functional residual capacity (FRC) is currently available on some ventilators. We report a case wherein the FRC measurement tool was used to identify lung volume changes associated with pulmonary complications.

## Case Report

A 35-year-old woman with a diagnosis of Guillian Barré Syndrome received mechanical ventilation in a Bilevel mode, using Engstrom Care Station Ventilator^®^ (GE Datex, Madison, Wisconsin USA). The mode was later changed to pressure support ventilation (PSV) with a positive end expiratory pressure (PEEP) of 5 cm H_2_O and a pressure support (PS) of 15 cm H_2_O. Her dynamic respiratory compliance (C_rs_) was 29 ml/cm H_2_O. With improvement in muscle power, extubation of the trachea was attempted on the 14^th^ day, which failed due to severe laryngeal edema. Reintubation and mechanical ventilation for one day was followed by spontaneous breathing on T piece.

A day later, lower respiratory tract infection necessitated reinstitution of mechanical ventilation in PSV mode (PEEP of 5 cm H_2_O and PS of 15 cm H_2_O). The patient continued to be tachypneic with an SpO_2_ of 87% on an FiO_2_ of 0.5. Her C_rs_ at this time was 23 ml/cm H_2_O and FRC was 940 ml. This loss of lung volume was evident on chest radiograph (left lower lobe collapse and diffuse infiltration of the right lung). The PEEP was increased to 10 cm H_2_O and 15 minutes later the FRC increased to 1088 ml. Since this improvement was not adequate, the PEEP was further increased to 20 cm H_2_O for 40 seconds. This technique, in many respects, is similar to the extended sigh used by Constantin *et al*,[2] for lung recruitment, but the highest PEEP level used for recruiting maneuver was lower and its duration of application was shorter (40 seconds in our patient as against 15 minutes in Constantin’s study). FRC, however, improved with this, and 2 hours later, the PaO_2_ /FiO_2_ increased to 306.

**Figure 1:**
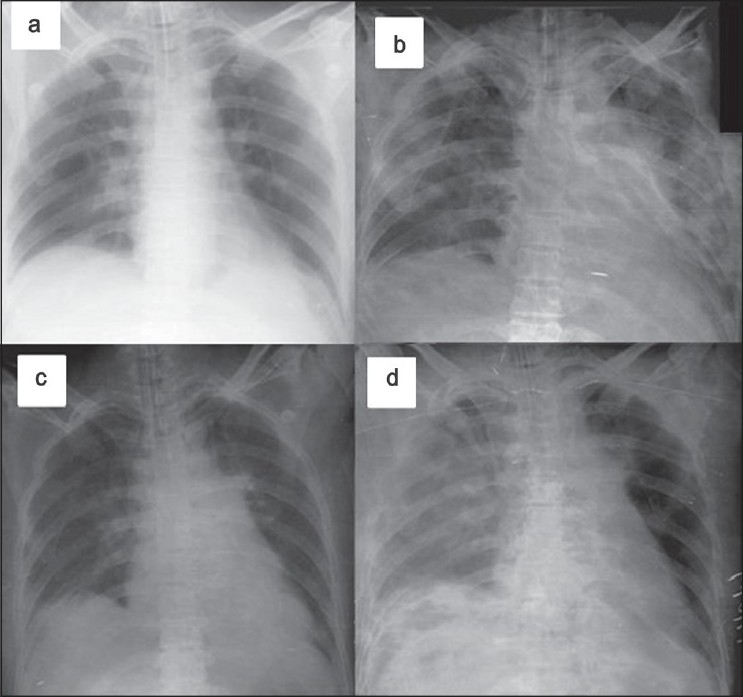
Chest radiographs of the patient. (a) Normal chest radiograph on Day 15, (b) Left lower lobe collapse and infiltration on Day 16, (c) Two hours after recruitment on Day 16, (d) Infiltration of right lung on Day 17.

One day later, the patient’s chest radiograph revealed extension of infiltrates on the right lung. Lung compliance and FRC also decreased significantly. Microbiology revealed methicillin-resistant *Staphylococcus*, which was treated with vancomycin. Forty-eight hours later, there was a significant clearance of pulmonary crepitations and reduction of infiltrates on chest radiograph. This was also accompanied by a considerable improvement in compliance and FRC. Table 1 shows the details of ventilatory settings, C_rs_ and FRC at different stages during the course of illness and Figure 1 shows chest radiographs corresponding to the significant events.

**Table 1:** Respiratory parameters during the ICU course of the patient

Day of illness	Ventilatory mode	PEEP (cm H2O)	Peak pressure setting (cm H_2_O)	Crs	FRC (ml)	pH	PaCO_2_ (mmHg)	PaO_2_/FiO_2_ (mmHg)	Comment
15	PSV	8	15	29	2266	7.4	33.3	290	One day post-reintubation after failed extubation
16	PSV	5	15	23	940	—	—	SaO_2_ 87%*	Two days post-reintubation after failed extubation; left lower lobe collapse on X-ray chest
16	PSV	10	15	27	1088	—	—	SaO_2_ 95%*	15 minutes after increasing the PEEP from 5 to 10 cm H2O
16	PSV	10	15	28	1142	—	—	SaO_2_ 100%*	10 minutes after a recruitment maneuver done for 40 seconds
16	PSV	8	15	32	1845	7.54	27.3	306	2 hours post-recruitment
17	PSV	10	15	19	997	7.48	36.0	180	Right lung infiltrates
19	PSV	10	15	29	2178	7.50	36.2	290	Significant improvement in ventilation on the chest radiograph

PEEP = Positive end expiratory pressure; Crs = Dynamic compliance; FRC = Functional residual capacity; PSV = Pressure support ventilation All blood gas values were obtained at an FiO2 of 0.5

* Arterial blood gas not available as the patient underwent emergent alveolar recruitment with SaO_2_ as a guide

## Discussion

FRC is an important determinant of oxygenation in ALI. Quantification of the loss of lung volume and the volume recruited in response to a recruitment maneuver are of practical importance. Radiology of the lung, pulmonary compliance measurement and blood gas analysis are commonly used to detect alterations in pulmonary function. Lung volume measurement based on spiral computerized tomography (CT) scans has been described earlier, but this requires transport of sick patients to the radiology suites.[3,4] Direct measurement of FRC using nitrogen washout is not practical for intensive care unit (ICU) patients.

The bedside FRC measurement tool is based on the principle of nitrogen washout, where the fractional nitrogen concentration in a gas mixture is calculated by subtracting the fractional concentrations of O_2_ and CO_2_. The N_2_ concentration of gases in the FRC of the lung is measured at a baseline FiO_2_ and after washing a part of the N_2_ by increasing the FiO_2_ by 10%. From these values of N_2_ concentration and the volume of N_2_ washed out (calculated by integrating the difference in FN_2_ in the expired and inspired gases over a given number of breaths), it is possible to measure the FRC values. Details of the technique are described in an earlier publication.[5]

The present case illustrates the value of the FRC tool in rapid quantification of the lung volume changes. Repeated bedside FRC measurements correlated with radiological findings and blood gas reports. The beneficial effects of ventilatory changes were reflected in the FRC measurements. FRC measurements increased the objectivity of our therapeutic interventions.

Recruitment maneuvers applied early during the course of acute respiratory distress syndrome ARDS prove more effective in improving the gas exchange and rapid resolution of pulmonary edema.[6,7] The improvement in our patient may be the result of early application of the recruitment maneuver. FRC measurement in this study was performed on spontaneous mode. Earlier studies have proven that FRC can be determined with good repeatability in patients during partial ventilatory support.[8]

Open lung tools available on some ventilators help to optimize the PEEP. If recruitment is carried out with monitoring of compliance or V_D_/V_T_, it is helpful in identifying the appropriate setting of PEEP. If the lung is substantially recruited with the recruitment maneuver, the peak pressure for a given tidal volume may decrease. Alternatively, if the patient is being ventilated in a pressure-controlled mode, there may be a scope for decreasing the pressure control level. The actual volume of lung recruited may be quantified by spirodynamic curves present in some ventilators.

FRC tool gives only a global measurement and does not differentiate lung recruitment from overdistension. PEEP applied after recruitment prevents recollapse of alveoli. However, too high levels of PEEP cause overdistension of the lung and increase the dead space ventilation. An optimal level of PEEP is one that prevents re-collapse, but avoids overdistension, while optimizing lung mechanics at minimal dead space ventilation. FRC, compliance, arterial oxygenation, and dead space fraction are the parameters that are theoretically helpful in choosing optimal PEEP. Maisch *et al*, showed that of these four parameters, compliance and dead space fraction are more suitable than the others. FRC and PaO_2_ are insensitive to alveolar overdistension.[9]

To conclude, FRC tool helps the ICU physicians to quantify the loss of lung volume in diseased lung and increase in the volume in response to recruiting measures. Future studies should evaluate, in large series, how well the FRC tool could complement the other measures in rapidly optimizing oxygenation in patients with pulmonary complications.
